# Design of a Sensitive Fluorescent Zn-Based Metal–Organic Framework Sensor for Cimetidine Monitoring in Biological and Pharmaceutical Samples

**DOI:** 10.1021/acsomega.2c00874

**Published:** 2022-06-21

**Authors:** Zahra Afravi, Valiollah Nobakht, Nahid Pourreza, Matineh Ghomi, Damian Trzybiński, Krzysztof Woźniak

**Affiliations:** † Department of Chemistry, Faculty of Science, Shahid Chamran University of Ahvaz, IR 6135743337 Ahvaz, Iran; ‡ Biological and Chemical Research Centre, Department of Chemistry, University of Warsaw, Żwirki i Wigury 101, 02-089 Warszawa, Poland

## Abstract

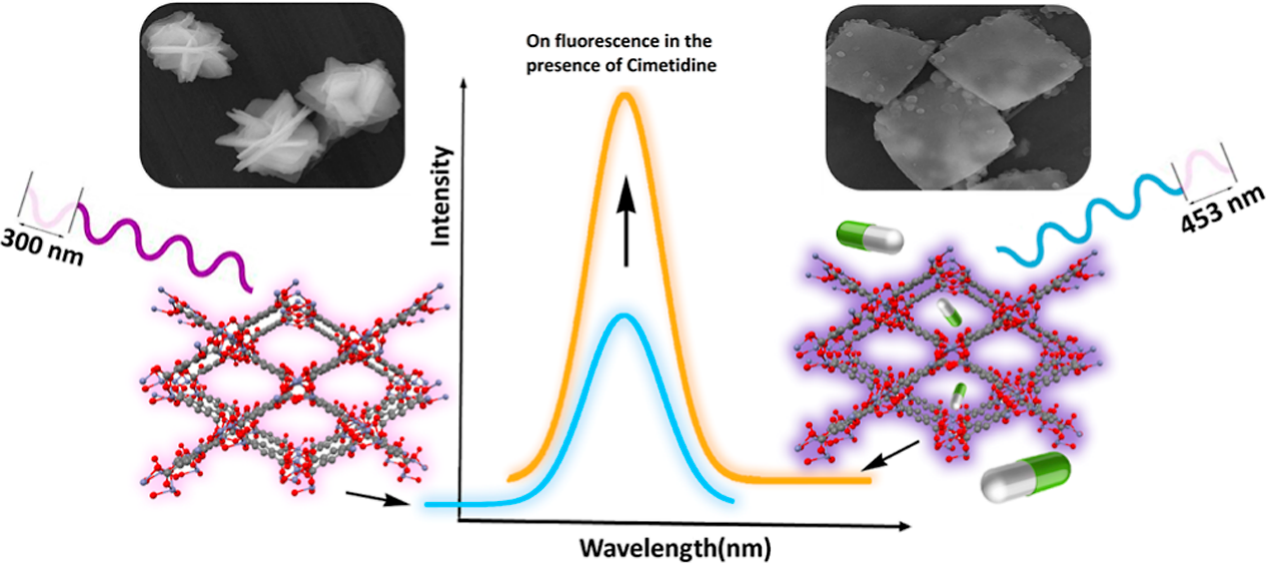

A new highly fluorescent zinc–organic framework [Zn_2_(btca)(DMSO)_2_]_n_ (**Zn-MOF**) was prepared via in situ ligand formation by the solvothermal reaction of Zn(NO_3_)_2_·6H_2_O and pyromellitic dianhydride (PMDA) in DMSO solvent. During the solvothermal reaction, PMDA was gradually hydrolyzed to a pyromellitic acid, 1,2,4,5-benzene tetracarboxylic acid (H_4_btca), to provide a tetracarboxylic acid as a linker in the reaction medium. Single-crystal X-ray diffraction analysis exhibits a 3D porous structure with open tetragonal channels running along the crystallographic *c*-axis. The **Zn-MOF** was explored as an on-mode fluorescent sensor for tracing cimetidine in biological fluids and pharmaceutical samples in the presence of interfering species. The results show a quick response in a short time range. The characteristics of this sensor were investigated by field-emission scanning electron microscopy, dynamic light scattering, energy-dispersive X-ray analysis, powder X-ray diffraction, Fourier transform infrared and UV–vis spectroscopy as well as thermogravimetric, and elemental analyses.

## 1 Introduction

Currently, people around the world have better living conditions through medical treatments, and a healthy life can be achieved through pills, syrups, and so forth. Medications often have the ability to cure diseases. Efforts in the field of medicine are focused on finding the best treatment for diseases, to the extent that medical research methods are mainly aimed at neutralizing the side effects of medical drugs and maintaining health in human life.^1^

The sensitive and selective detection of drugs in biological samples is attracting immense attention due to their pharmacodynamics and pharmacokinetics in human bodies.^2^ Researchers have reported that cimetidine causes many unwanted effects when it is administered at higher therapeutic doses.^3^ Cimetidine, *N*″-cyano-*N*-methyl-*N*′-[2([(5-methyl-1*H*-imidazole-4-yl)methyl]thio)ethyl]guanidine, is the prototype of the histamine H2-receptor antagonist among the most commonly prescribed drugs in the world,^4^ which can also be obtained without a prescription. It is widely used in clinical treatments for gastric reflux or other gastrointestinal diseases.^5,6^ The action of histamine on all H2-receptors prevents the production of acid in the stomach wall cells and improves gastric and duodenal ulcers. These two ulcers are one of the most common causes of indigestion, and cimetidine is effective in managing gastric hypersecretion.^7^ Cimetidine is safe enough that it is available without a prescription. However, there is a concern about cimetidine-induced neutropenia, and it can cause overdose or drug interactions. Serious side effects of cimetidine include confusion and hallucinations, decreased white blood cell counts, irregular heartbeat, and so forth; these effects may be more likely to occur in older adults and those who are ill or debilitated. Many drugs may affect cimetidine or be made less effective when taken at the same time as cimetidine. This includes prescription and over-the-counter medicines, vitamins, and herbal products.^8^ Thus, a study to evaluate and track cimetidine is felt necessary to solve this issue.

Various methodologies have been reported for detection of this drug; however, most of them are time-consuming, tedious, and require expensive instruments.^9−12^ Hence, detection studies and monitoring of cimetidine are imperative for improving drug therapy.

On the other hand, many efforts in the field of crystal engineering are under process to obtain new crystalline materials with desired properties. Crystal engineering is one of the amazing research fields in chemistry which combines chemistry and art to rationally design and create new structural architectures with desired properties and applications.^13^ In this regard, an explosion of interest in the design and synthesis of coordination polymers (CPs) and its unique subclass, that is, metal–organic frameworks (MOFs), has occurred over the past few decades due to their outstanding properties and a myriad of possible applications, which include traditional uses of microporous materials, such as gas storage, separation, and catalysis,^14−16^ as well as new realms in biomedicine,^17,18^ electronic devices, and information storage.^19,20^ MOFs are a fascinating class of porous crystalline substances that are composed of multidentate organic linkers and inorganic metal nodes that form exciting three-dimensional frameworks.^21,22^ Among the various categories of organic linkers, used in the structure of MOFs, multidentate carboxylate ligands are one of the most successful category of organic linkers which provide crystalline structures with suitable chemical stability.^23−25^ One of the most important goals of MOF synthesis has been to gain high-quality single-crystals for understanding various structural features of such materials. Because of the fast reaction between metal and ligand sources, obtaining a high-quality single-crystal is still a time-consuming and challenging step in the MOF synthesis process. To fix the problem, some crystallization methods, such as solvent layering and crystallization in gels,^26−28^ as well as in situ formation of starting materials which reduce the combination rate of the reagents can be applied.^29,30^ In this regard and in continuation of our research activity on the exploration of effective factors on the design, synthesis, and potential applications of new CPs,^31−33^ in this work, in situ ligand formation and subsequent MOF crystallization in a one-pot reaction has been examined. In this approach, a tetracarboxylate linker, 1,2,4,5-benzene tetracarboxylic acid, has been gradually obtained via hydrolysis of pyromellitic dianhydride and formed a new zinc(II)-based MOF crystal under solvothermal conditions. The as-prepared [Zn_2_(btca)(DMSO)_2_]_*n*_ (Zn-MOF) acts as an attractive recognition sensor for cimetidine determination. As far as we know, there are no reports on the sensing property of any MOFs for selective cimetidine detection. This MOF-based sensor illustrates good potential for detecting the drug cimetidine that exhibits no inherent fluorescence or color. The fluorescence intensity of [Zn_2_(btca)(DMSO)_2_]_*n*_ was enhanced in the presence of cimetidine (on-mode), and this fluorescence intensity variation was chosen as a monitoring signal. Flower-like aggregated square plates of [Zn_2_(btca)(DMSO)_2_]_*n*_ exhibited low fluorescence intensity due to external interactions between square plates, but the presence of cimetidine can separate the sheets of the MOF and increase the fluorescence intensity. Hence, the designed sensor can quantitatively recognize and monitor cimetidine in biological fluids (plasma and urine samples) and tablets.

## 2 Experimental Section

### 2.1 Chemicals and Instruments

All chemicals were of analytical grade or commercially available and used as received without further purification. Zn(NO_3_)_2_·6H_2_O (98%) and the pyromellitic dianhydride (97%) were purchased from Sigma-Aldrich. Dimethyl sulfoxide (DMSO) (99.9%) and anhydrous ethanol (95%) were purchased from Merck Chemical Co. Phosphate buffer, pH 6 was prepared by the addition of sodium hydroxide (0.1 mol L^–1^) to a phosphoric acid solution (0.1 mol L^–1^). The drug cimetidine was purchased from Sigma-Aldrich (St. Louis, MO, USA). A stock solution (10 ng mL^–1^) of cimetidine was prepared, and working solutions were prepared from the appropriate dilution of this stock solution. Milli-Q-water (Cell Culture Grade, Bioidea Company, Iran) was utilized to prepare the solutions.

The vibrational spectra in the infrared region were recorded from KBr pellets using a PerkinElmer Spectrum Two Fourier transform infrared (FT-IR) spectrometer in the region of 400–4000 cm^–1^. The amounts of C, H, N, and S were determined by elemental analysis with an Elementar Vario EL CHNS analyzer. The powder X-ray diffraction (PXRD) pattern of the sample was measured on a Philips X’Pert Pro diffractometer operating at 40 kV voltage and 30 mA current with CuKα X-ray radiation (λ = 1.54184 Å). Thermogravimetric analysis (TGA) was performed by TA-TGA Q5000IR thermal analysis equipment under a nitrogen flow of 40 mLmin^–1^. The heating rate was about 10 °C min^–1^ within a temperature range of 25–600 °C. The fluorescence spectra were recorded by a Hitachi F-7000 spectrofluorometer (Japan) equipped with a xenon lamp source. The bandwidths of excitation and emission wavelengths were set at 10 and 5 nm, respectively. The UV–visible absorption spectra were recorded using a Jenway spectrophotometer model 6705 (UK). The pH adjustments were performed using a digital pH-meter model 632 (Metrohm, Herisau, Switzerland). Field-emission scanning electron microscopy (FE-SEM) images have been taken by the MIRA3 model. Dynamic light scattering (DLS) was obtained by Nano ZS (red badge) ZEN 3600, Malvern Company (England). Simulated PXRD patterns, based on single-crystal X-ray diffraction data, were prepared using Mercury software.^34^ Topological analysis was performed using ToposPro.^35^

### 2.2 Synthesis of **Zn-MOF**

#### In Situ Synthesis

To prepare Zn-MOF via in situ ligand formation from pyromellitic dianhydride hydrolysis, a solution of Zn(NO_3_)_2_·6H_2_O (0.292 g, 0.98 mmol) in DMSO (5 mL) was added to a solution of pyromellitic dianhydride (0.118 g, 0.54 mmol) in DMSO (18 mL). The resulting mixture was transferred into a 40 mL Teflon-lined stainless-steel autoclave reactor and then heated at 120 °C for 72 h. Then, slowly cooled to room temperature at a rate of 6 °C h^–1^. Colorless crystals, suitable for single-crystal X-ray diffraction analysis were isolated, washed with DMSO and anhydrous ethanol to remove all impurities, and dried in air. Yield: ca. 75% (based on Zn). Anal. Calcd for C_14_H_14_O_10_S_2_Zn_2_ (FW = 537.11 g mol^–1^): C, 31.30; H, 2.60; S, 12.14%; found: C, 30.86; H, 2.52; S, 12.14%.

#### Direct Synthesis

A solution of Zn(NO_3_)_2_·6H_2_O (0.292 g, 0.98 mmol) in DMSO (5 mL) was added to a solution of pyromellitic acid, H_4_btca, (0.137 g, 0.54 mmol) in DMSO (18 mL). The resulting mixture was transferred into a 40 mL Teflon-lined stainless-steel autoclave reactor and then heated at 120 °C for 72 h. Then, slowly cooled to room temperature at a rate of 6 °C h ^–1^. Colorless crystals were collected, washed with DMSO and anhydrous ethanol to remove all impurities, and dried in air. Yield: ca. 55% (based on Zn).

### 2.3 Single-Crystal Structure Determinations

Good quality single-crystals of **Zn-MOF** were selected for the X-ray diffraction experiments at *T* = 100(2) K. The diffraction data were collected on the Agilent Technologies SuperNova double source diffractometer with MoKα (λ = 0.71073 Å) radiation, using CrysAlis RED software.^36^ The analytical absorption correction using a multifaceted crystal model based on expressions derived by R.C. Clark & J.S. Reid^37^ implemented in SCALE3 ABSPACK scaling algorithm, was applied.^36^ The structural determination procedure was carried out using the SHELX package.^38^ The structure was solved with direct methods and then successive least-square refinement was carried out based on the full-matrix least-squares method on *F*^2^ using the SHELXL program.^38^ All H-atoms were positioned geometrically with the C–H equal to 0.93 and 0.96 Å for the aromatic and methyl H-atoms, respectively, and constrained to ride on their parent atoms with *U*_iso_(H) = *xU*_eq_(C), where *x* = 1.2 for the aromatic and 1.5 for the methyl H-atoms, respectively. The DMSO molecule containing the S1 atom was subject to the RIGU restraint. The figures for this publication were prepared using Olex2,^39^ Mercury, and ToposPro.

### 2.4 On-Fluorescence Assay of Cimetidine

The typical experiment is described as follows: 2 mg of **Zn-MOF** and 0.1 mL phosphate buffer pH 6 was dispersed into 5 mL milli-Q water via ultrasonication for 20 min. The resulting mixture was considered as a blank and its fluorescence spectrum was recorded at an excitation wavelength (λ_ex_) of 300 nm. The emission wavelengths were registered from 350 to 600 nm and the maximum emission intensity (λ_em_) was noted as the fluorescence intensity of the blank (*F*_0_). All fluorescence spectra were carried out with the slit widths of 10 and 5 nm for the excitation and emission wavelengths, respectively. For the determination of cimetidine, various concentrations of cimetidine (final concentration in the range of 1–10 ng mL^–1^) were added to the designed sensor so that the alterations in the final volume were ignored. The highest intensity of emission wavelengths in the presence of cimetidine was recorded and defined as *F*. The fluorescence “turn-On” intensity of the sensor was measured after 3 min. The sensor responses were described as the ratio of *F*/*F*_0_.

### 2.5 Pretreatments of Real Samples

Two fresh human plasma and urine samples were obtained from healthy volunteers of the medical diagnosis clinic. Trichloroacetic acid solution was added to the plasma solution in a volume ratio of 2:1, centrifuged, and filtered to precipitate and remove all proteins from the plasma.^40^ 1 mL of urine sample was only diluted up to three times without further pretreatment.

Two commercial cimetidine tablets (Kimia Darou Pharmaceutical Company) and ampule (Exir Company) were prepared and the desired pretreatment was performed before analysis. The tablet was completely milled and dissolved into the minimum amount of required Milli-Q water. After vigorous shaking for 5 min, the resulting solution was passed through a filter paper. The filtrate was transferred into a 25 mL volumetric flask, diluted to the mark, and saved as a stock tablet solution. The sequential dilutions were performed to prepare the appropriate stock solution of cimetidine ampoule. The desired concentrations of cimetidine within the linear range of analytical methodology were obtained through the proper dilution. Finally, all real samples were tested according to the suggested procedure.

## 3 Results and Discussion

### 3.1 Synthesis of the **Zn-MOF** Sensor

From the reaction of Zn(II) ions with pyromellitic dianhydride as a source of tetracarboxylate linker, 1,2,4,5-benzene tetracarboxylic acid (H_4_btca), under solvothermal conditions, a new 3D MOF [Zn_2_(btca) (DMSO)_2_]_n_ (**Zn-MOF**) has been successfully prepared. One of the barriers to the faster growth of novel as-prepared MOF structures is the challenge of growing MOF single-crystals of quality suitable for structural determination using a single-crystal X-ray diffractometer. In this context, fast reaction of starting reagents has often led to the appearance of a precipitate instead of well-grown single-crystals. Several different crystallization methods have been introduced to reduce the combination rate of metal ions and linker ligands such as solvent layering, crystallization in gels,^28^ and so forth. Another amazing approach is in situ gradual formation of metal ions or linker ligands in the reaction medium. In this approach, metal ions can be produced from insoluble metal oxides or pure metal electrodes and carboxylate linker ligands can be generated in situ via the hydrolysis of anhydride species. The hydrolysis reaction is promoted in the presence of water and transition-metal ions as a catalyst.^41−43^ With this idea in mind, pyromellitic dianhydride, as the source of the linker ligand, has been reacted with a Zn(II) ion under solvothermal conditions for the in situ gradual formation of 1,2,4,5-benzene tetracarboxylic acid linker (H_4_btca) via gradual hydrolysis and crystallization of the desired **Zn-MOF**. The as-prepared MOF is insoluble in water and all common organic solvents, stable toward air and moisture, and well characterized by FT-IR spectroscopy, thermogravimetric, and elemental analyses, PXRD, and single-crystal X-ray diffraction analysis.

### 3.2 Characterization of the **Zn-MOF** Sensor

**Figure 1:**
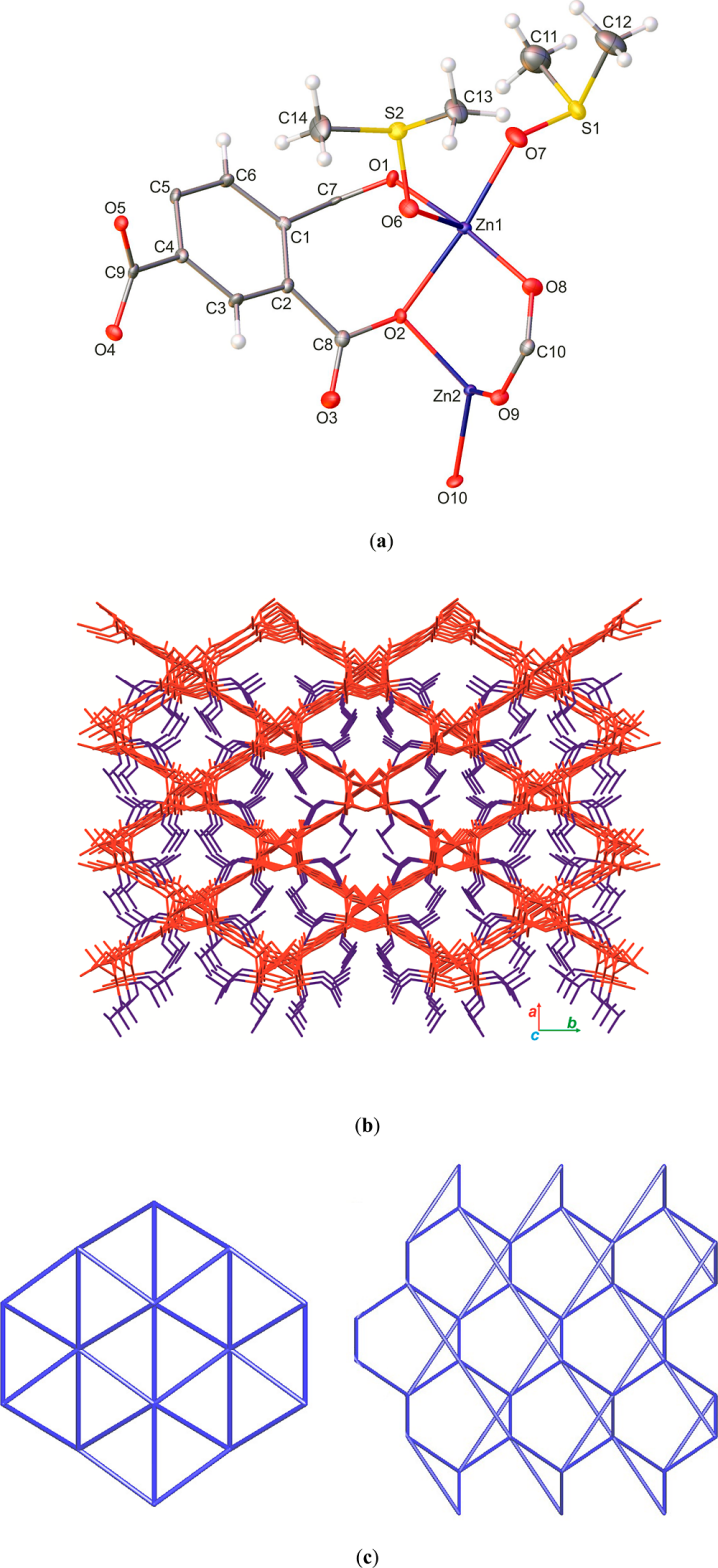
Crystal structure of [Zn_2_(btca)(DMSO)_2_]_*n*_ (**Zn-MOF**). (a) Asymmetric part of the unit cell of the crystal lattice with the atom labeling. The displacement ellipsoids are drawn at the 50% probability level and the H atoms are shown as small spheres of arbitrary radius; (b) general view on the 3D framework along the *c*-axis with open rhomboidal channels (colored in red), occupied by the DMSO molecules (colored in blue). The H-atoms were omitted for clarity; (c) topological view of the simplified **sda** net along the *a*- (left) and *c*- (right) axes.

Single-crystal X-ray diffraction analysis reveals a three-dimensional polymeric structure for [Zn_2_(btca)(DMSO)_2_]_*n*_. The investigated compound crystallizes in the monoclinic *Cc* space group with *Z* = 4. Crystallographic data and refinement details of the complex [Zn_2_(btca)(DMSO)_2_]_*n*_ are gathered in Table 1 and its crystal structure is shown in Figure 1.

**Table 1:** Crystal Data and Structure Refinement Details for **Zn-MOF**

empirical formula	C_14_H_14_O_10_S_2_Zn_2_
formula weight	537.11
temperature/K	100(2)
crystal system	monoclinic
space group	*Cc*
*a*/Å	10.44615(15)
*b*/Å	15.5309(2)
*c*/Å	11.69200(18)
α/°	90
β/°	93.0473(14)
γ/°	90
volume/Å^3^	1894.21(5)
*Z*	4
ρ_calc_ g/cm^3^	1.883
μ/mm^–1^	2.804
*F*(000)	1080.0
crystal size/mm^3^	0.20 × 0.11 × 0.09
radiation	MoKα (λ = 0.71073 Å)
2Θ range for data collection/°	4.704 to 52.724
index ranges	–13 ≤ *h* ≤ 13, −19 ≤ *k* ≤ 19, −14 ≤ *l* ≤ 14
reflections collected	38929
independent reflections	3869 [*R*_int_ = 0.0490, *R*_sigma_ = 0.0251]
data/restraints/parameters	3869/26/257
goodness-of-fit on *F*^2^	1.053
final *R* indexes [*I* ≥2σ (*I*)]	*R*_1_ = 0.0236, w*R*_2_ = 0.0584
final *R* indexes [all data]	*R*_1_ = 0.0241, w*R*_2_ = 0.0588
largest diff. peak/hole/e Å^–3^	0.86/–0.69
flack parameter	–0.007(5)

The asymmetric unit contains two Zn^2+^ ions, a fully deprotonated btca^4–^ ligand, and two coordinated DMSO guest molecules (Figure 1a). There are two crystallographically independent Zn(II) ions in the crystal structure. The Zn1 ion shows a distorted octahedral geometry, coordinated by four O atoms from three discrete btca^4–^ ligands (O1, O2, O5, and O8) and two O atoms from DMSO molecules (O6 and O7). Due to the rigidity of chelating carboxylate groups, a coordination environment of the Zn1 centers is distorted from the ideal octahedral geometry with a bond angle in the range 80.27(12)–94.06(13)°. Interestingly, the cis-coordinated DMSO molecules are orientated in a way such that the angle between their coordinated O atoms is lower than 90° [O6–Zn1–O7 87.08(13)°]. The Zn1–O bond lengths range from 2.055(3) to 2.180(3) Å. The shortest bond lengths of 2.055(3) and 2.066(3)° correspond to the coordinated DMSO molecules, while the longest one [Zn1–O2 2.180(3) Å] is attributed to the O2 atom of the carboxylate moiety, which exhibits a bridging– chelating mode (Figure 1a). The Zn2 shows a highly distorted tetrahedral geometry, coordinated by four O atoms from four btca^4^– anions. The value of deviation of metallic center can be calculated by the τ_4_ Houser value.^44,45^ The values of this parameter for perfect tetrahedral and trigonal pyramidal geometries are 1.00 and 0.85, respectively, and for the seesaw geometry, it varies from about 0 to 0.64. The calculated τ_4_ value of 0.75 shows a geometry between seesaw and trigonal pyramidal for the Zn2 centers. The Zn2–O bond lengths are in the range from 1.959(3) to 1.996(3) Å. Fully deprotonated tetracarboxylate ligands in the form of btca^4–^ anions act as a μ_6_-bridging moiety in the structure of the complex. Four carboxylate groups of the ligands show three different coordination modes with Harris notations^46^ of 2.11 and 2.20 (Figure S1). The presence of four carboxylic groups in the structure of H_4_btca, which can be partially or completely deprotonated, leading to a wide variety of coordination modes in the polymeric structures.^47,48^ Some of the common coordination modes are shown in Figure S2.

Extended connection of Zn(II) centers with btca^4–^ linkers in the crystal forms a three dimensional MOF with open tetragonal channels running along the ***c***-axis (Figures 1b and S3).

The channels are occupied by the DMSO molecules coordinated to the octahedral zinc centers (Figure 1b). A non-classical C–H•••O (H•••O 2.59 Å) hydrogen bond was also observed between an H atom of a DMSO molecule and O12 atom of the linker. The coordination bond and hydrogen interactions between the DMSO molecules and the skeleton of the framework increase the immobilization of the solvent molecules into the pores of the structure. Excluding the solvent molecules from the above-mentioned channels, it was possible to predict a potential solvent accessible volume of about 48% for this specific structure.^49^ To better understand the complex 3D structure of **Zn-MOF**, topological analysis by the ToposPro program was also performed. A simplification of the investigated structure gives a uninodal six-connected net with a {3^3^.5^9^.6^2^.7} point symbol and **sda** topology^50,51^ for the compounds (Figure 1c).

The FT-IR spectrum of **Zn-MOF** (Figure S4) shows two strong sharp bands in the ranges of 1350–1450 and 1538–1625 cm^–1^, assigned to the symmetric and asymmetric stretching vibrations of coordinated carboxylate groups, respectively.^25^ The observed band with weak to medium intensity at 1050 cm^–1^ can be assigned to the S=O vibrations of the coordinated DMSO molecules. The peaks in the region 2900–3100 cm^–1^ can be attributed to the aromatic and aliphatic −C–H vibration bands of the btca^4–^ and DMSO components. The strong broad band at 3432 cm^–1^ can be attributed to the stretching vibrations of the adsorbed water molecules.

The powder X-Ray diffraction was used to check the purity of the bulk **Zn-MOF** samples. The PXRD patterns of the in situ synthesis as well as the direct formation of the **Zn-MOF** via the reaction of pyromellitic acid (H_4_btca) and Zn(II) ions are shown in Figure S5. The result shows that all the peaks displayed in the measured pattern at room temperature closely match those in the simulated patterns generated from single-crystal diffraction data, indicating single-phase purity and high crystallinity of the products of both synthetic methods. In addition, this result reveals for the MOFs with low solubility that the reaction product is always a powder instead of suitable single-crystals, the gradual in situ ligand formation is an alternative approach to grow suitable single-crystals for X-ray crystallography.

To examine the thermal stability, TGA for the **Zn-MOF** was also performed on polycrystalline samples under a nitrogen atmosphere from 25 to 600 °C with a heating rate of 10 °C.min^–1^ (Figure S6). The TGA curve shows a weight loss of 29.0% up to about 320 °C, ascribed to the departure of two coordinated DMSO molecules. Upon further heating from 400 to 500 °C a weight loss of 45.0% was observed, which is consistent with the decomposition of the btca^4–^ organic linkers and a collapse of the 3D structure. A residual weight of about 15% is attributed to the formation of ZnO.

### 3.3 Fluorescent Behavior of the **Zn-MOF** Sensor

CP- and MOF-containing Zn(II) nodes and conjugated aromatic linkers are well-known photoactive materials.^52^ Due to the d^10^ electron configuration of Zn^2+^, the luminescence behavior of these materials can be presumed to the π–π* transition of the ligand. Upon complex formation, a red shift in the emission spectrum of the MOF, with respect to that of its free ligand, is observed. Because Zn(II) ions are difficult to oxidize or reduce, the emissions of Zn(II) MOFs are neither metal-to-ligand charge transfer nor ligand-to-metal charge transfer. Thus, the emission is probably attributed to the intraligand π–π* transitions, perturbed by metal coordination.^53,54^ In the Zn(II) carboxylate-based MOFs, the highest occupied molecular orbital (HOMO) is likely the π-bonding orbital from the aromatic rings and the lowest unoccupied molecular orbital (LUMO) is related mainly to the Zn–O (carboxylate) π*-antibonding orbital, which is localized often on the metal centers.^52,55^ As the luminescence of Zn(II) MOFs mainly originates from the π–π* transition of ligand and on the other hand, this transition is mostly affected by guest molecules and solvent polarity changes;^56^ hence, potential luminescence properties of the **Zn-MOF** are first investigated in various solvents.

**Figure 2:**
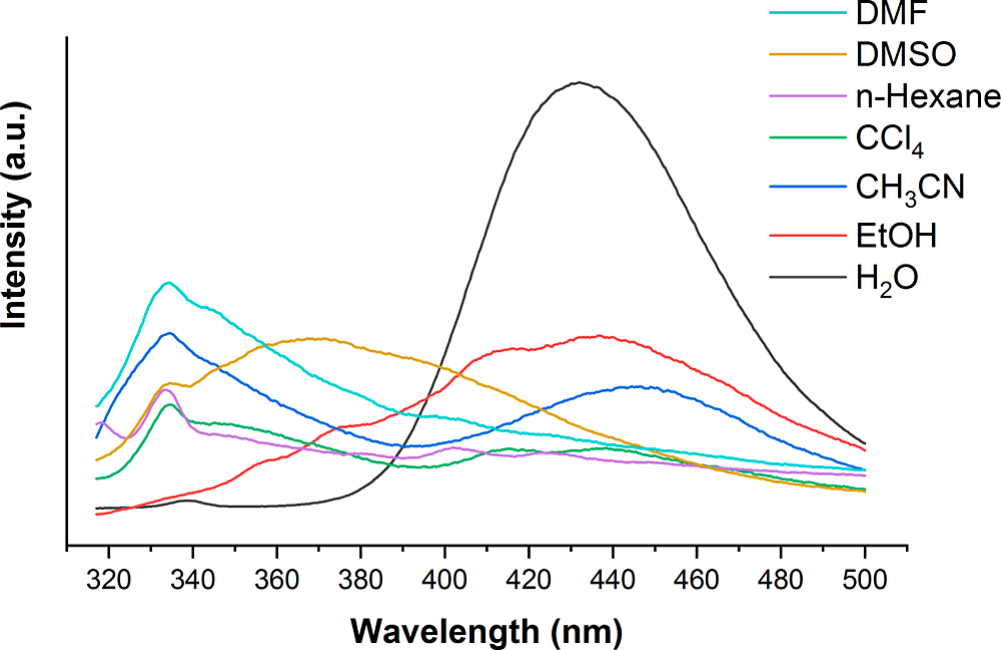
Emission spectra of the dispersed **Zn-MOF** in different solvents with various polarities.

The suspension of **Zn-MOF** in a solvent illustrates an emission peak upon the excitation at a wavelength of 300 nm. As expected, the emission peaks of the **Zn-MOF** are different in different solvents. The emission and UV–vis spectra as well as the emission values for the synthesized **Zn-MOF** in different solvents with various polarities are shown in Figures 2 and S7, and Table 2, respectively. As can be seen in Table 2, the maximum emission wavelengths of the **Zn-MOF** shift to shorter wavelengths in the presence of DMSO, DMF, *n*-hexane, and CCl_4_ while for more polar ethanol and H_2_O, solvents shift to longer wavelengths. As in this transition, the excited state (π*) is more polar than the ground state (π), the π* is stabilized relative to the ground state and shows a red shift in more polar solvents.^56^ Given the above, as well as the higher intensity of the fluorescent signal in water and the aqueous media of biological systems, the choice of water as a solvent for further studies seems to be reasonable. Therefore, H_2_O was selected as a suitable solvent for further analysis. The **Zn-MOF** emission peak in water can be considered as a fluorescence signal for monitoring the sensing process.

**Table 2:** Emission Values of **Zn-MOF** in Different Solvents with Their Emission/Absorption Wavelengths

solvent	λ_abs_ (nm)	λ_em_ (nm)	intensity
DMSO	318	367.5	1437.2
DMF	320	334.5	1695.6
CH_3_CN	309	334.5	1514.0
		446.5	1063.0
CCl_4_	469	335.0	915.6
*n*-hexane	385	333.5	816.0
EtOH	455	440.0	1609.0
H_2_O	**493**	**432.0**	**3650.0**

**Scheme 1:**
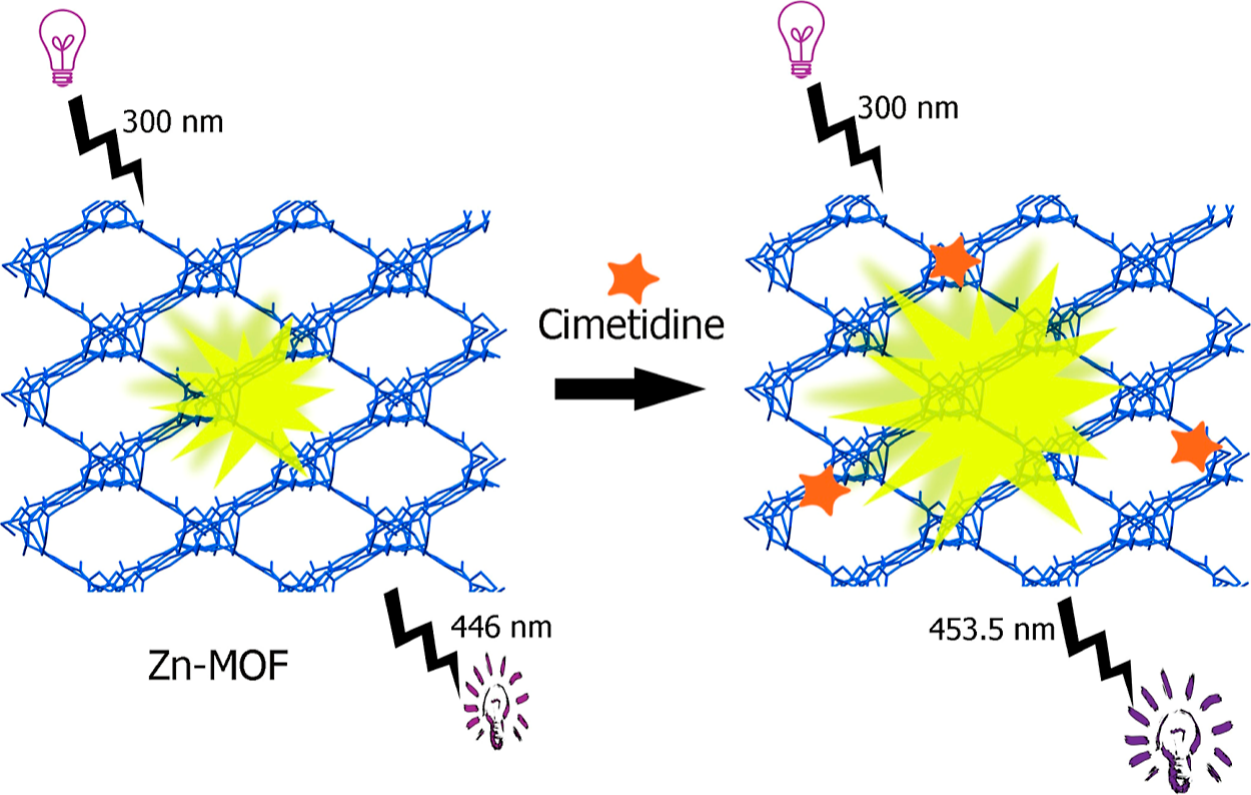
Increase of Fluorescence Intensity of the **Zn-MOF** in the Presence of Cimetidine (Turn-On Mode)

Interestingly, it was found that the fluorescence intensity of [Zn_2_(btca)(DMSO)_2_]_*n*_ is enhanced in the presence of cimetidine (Scheme 1).

**Figure 3:**
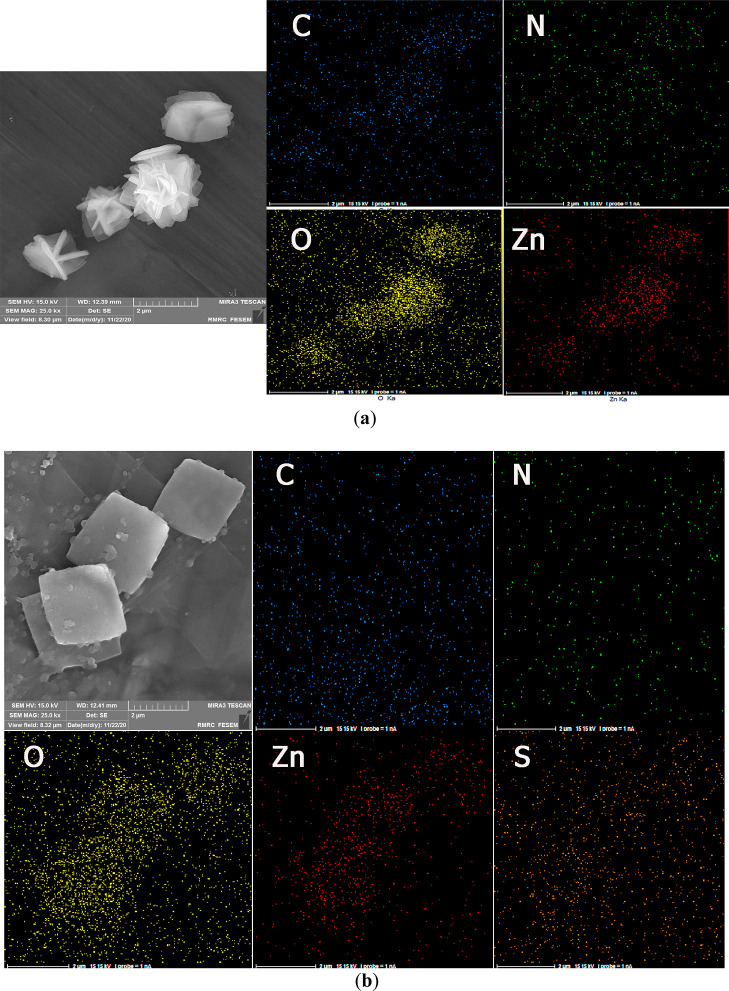
FE-SEM images and EDX elemental mapping for (a) **Zn-MOF** sensor and (b) **Zn-MOF**–cimetidine mixture.

The FE-SEM images of the **Zn-MOF** crystals in the absence and presence of cimetidine show interesting results. The images exhibit a flower-like morphology, consisting of aggregated square plates, for the as-synthesized **Zn-MOF** and a discrete square plate shape for the **Zn-MOF**-cimetidine mixture (Figure 3). In the presence of cimetidine, the flower-like structures disintegrate into single square plates. This observation may be due to the presence of stronger interactions between cimetidine molecules and the surface of **Zn-MOF** square plates with respect to the interactions between the adjacent **Zn-MOF** plates. The EDX elemental mapping of the **Zn-MOF** in the presence of cimetidine also showed the well distribution of sulfur atoms over the sample, which can be attributed to the presence of cimetidine drug.

**Figure 4:**
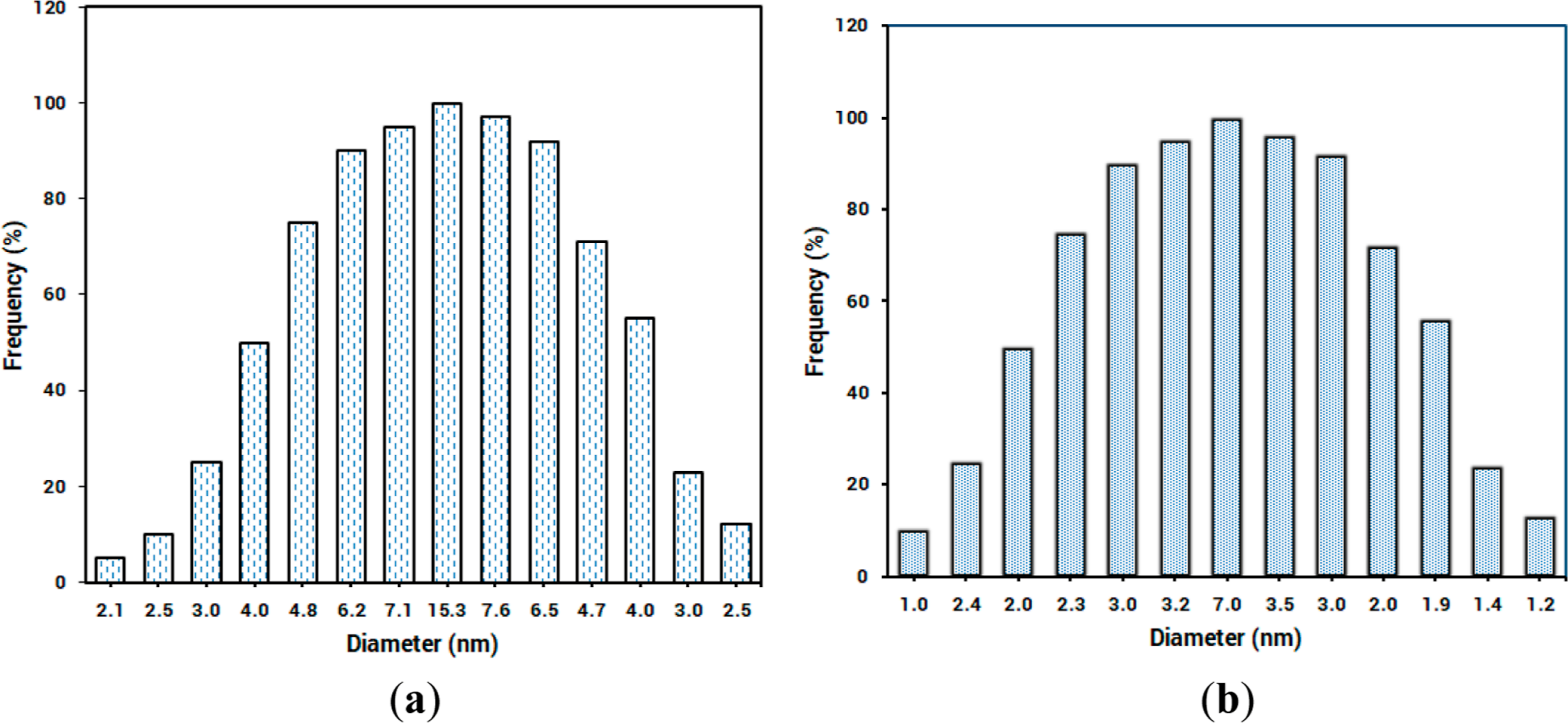
DLS diagrams for (a) **Zn-MOF** and (b) **Zn-MOF**-cimetidine mixture under optimal conditions.

The results of the DLS analysis also illustrate that the average size of MOF was almost halved in the presence of cimetidine. This average size decreased for 100% frequency from 15.3 (**Zn-MOF**) to 7.0 nm (**Zn-MOF**-cimetidine mixture) (Figure 4). The observation is consistent with disintegration of flower-shaped aggregations to single square plates in the presence of cimetidine in the SEM analyses.

**Figure 5:**
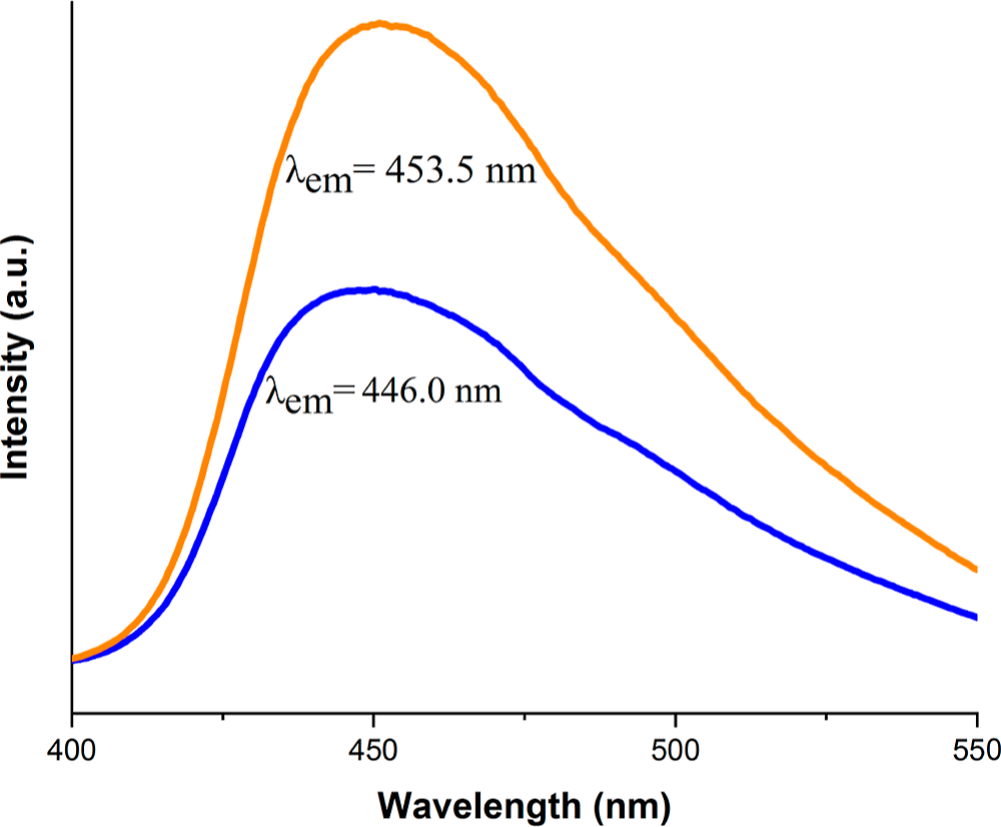
Emission spectra of the dispersed **Zn-MOF** in the absence (blue) and presence (orange) of cimetidine (50 ng mL^–1^) upon excitation at 300 nm in H_2_O.

Emission spectra of the dispersed **Zn-MOF** in distilled water before and after adding of cimetidine upon λ_ex_ = 300 nm are shown in Figure 5. An obvious enhancement in the emission intensity of this sensor was seen after adding cimetidine. These enhancements in the fluorescence intensity showed a linear relationship (in two concentration areas) with the increase of drug concentrations.

In general, electron-rich molecules are found to enhance the luminescence of the hosts.^52^ On the other hand, due to the presence of several functional groups in the structure of cimetidine, it can easily interact with surface of the MOF via hydrogen bonds. Hence, the observed enhanced luminescence intensity in the presence of cimetidine may be due to the antenna effect of the guest analyte that absorbs the excitation energy and transfers it to the LUMO of the **Zn-MOF**,^52^ resulting in enhanced emission intensity.

### 3.4 Effect of pH on the Sensor Performance

To investigate the influence of pH on sensor responses, the desired amount of hydrochloric acid (0.1 mol L^–1^) and sodium hydroxide (0.1 mol L^–1^) was added to the stated sensing procedure, and the desired pH was adjusted in the range of 3–11. As can be seen in Figure S8, the fluorescent response consequently increased in a range of 4.0–6.0. At a higher pH, the signals were falling and showed no significant responses. Hence, pH 6 was selected as the optimal one to achieve the best response.

Various buffers (acetate, citrate, phosphate, and Britton-Robinson buffers) were investigated at the optimal pH, and phosphate buffer exhibits the best response among others. Finally, 0.1 mL phosphate buffer pH 6 was used to adjust the pH through the recommended procedure.

### 3.5 Effect of Reaction Time

To have an estimate of the optimal conditions for sensing of cimetidine as an enhancer factor, various reaction times were investigated in the time domain from 1 to 7 min for three concentrations of cimetidine. As displayed in Figure S9, the emission intensities are enhanced along with increasing cimetidine concentrations. Besides, the emission intensity of the proposed sensor slightly increases after the reaction time reaches 3 min. Fluorescence intensity variations can be neglected over 3 to 7 min. Hence, 3 min was selected as an optimum time of reaction for further research.

### 3.6 Analytical Parameters and Performance

**Figure 6:**
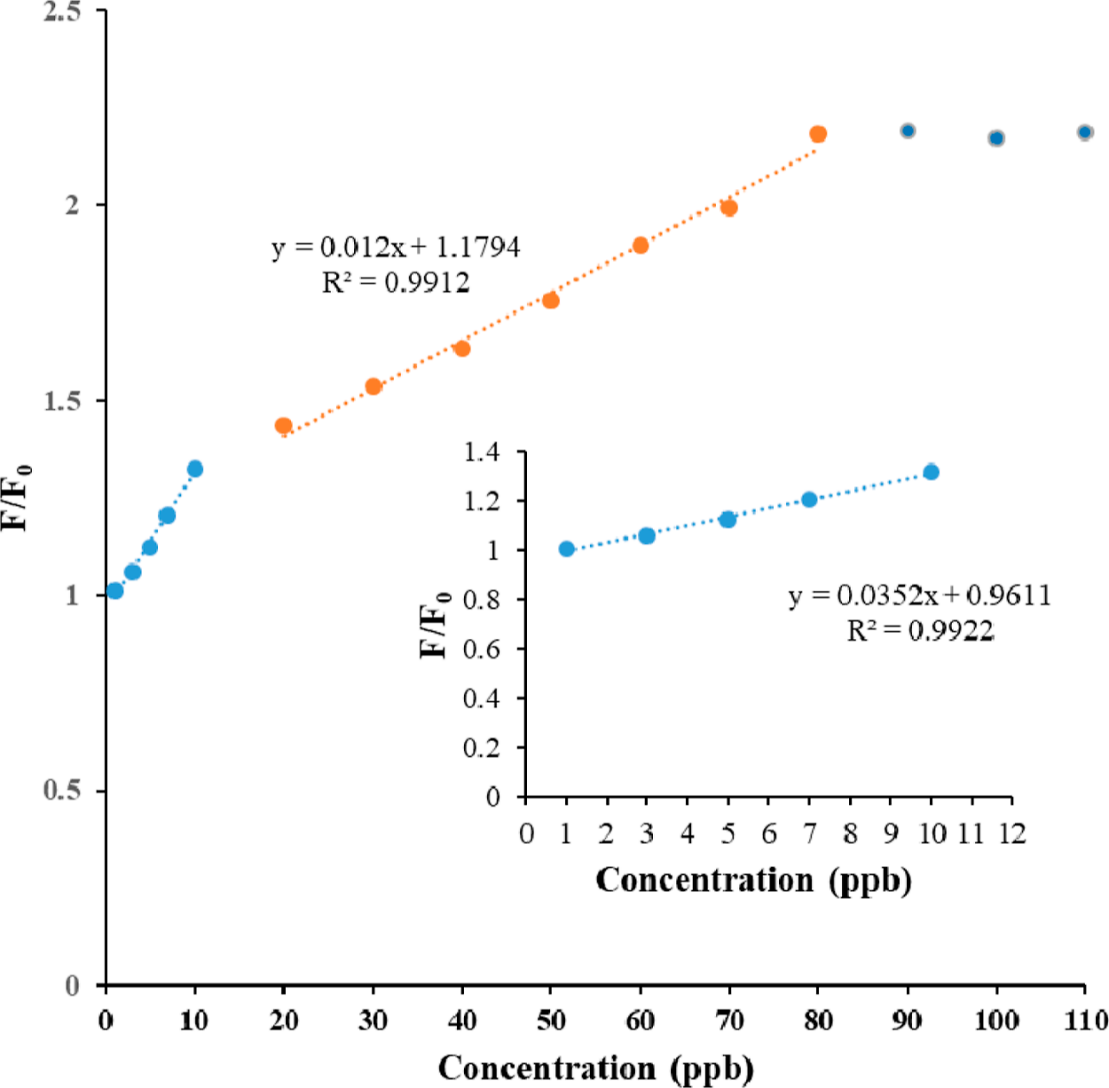
Linear relationships of **Zn-MOF** sensor in the presence of different concentrations of cimetidine under optimum conditions at two concentration areas, inset: the linear calibration graph for cimetidine sensing (1.0 to 10.0 ng mL^–1^).

The emission spectra of the **Zn-MOF** sensor in the presence of various concentrations of cimetidine indicated an obvious enhancement in the emission intensity of the sensor. Upon the addition of cimetidine to the **Zn-MOF** suspension in the optimized experimental conditions, the fluorescence intensities gradually increased with drug concentrations (Figure 6). The enhanced fluorescence intensity displayed two linear relationships at two concentration areas from 1.0 to 10.0 ng mL^–1^ and from 20.0 to 80.0 ng mL^–1^ (inset of Figure 6). The fitted linear equations would be expressed as *F*/*F*_0_ = 0.0352 [Cimetidine] + 0.9611 (*r*^2^ = 0.9922), *F*/*F*_0_ = 0.012 [Cimetidine] + 1.1794 (*r*^2^ = 0.9912), and the results show a good linear relationship.

Limit of detection (3Sd/*K*) was calculated based on the standard deviation (Sd) of 10 times measuring of *F*_0_ and the slope (*K*) of the calibration graph. The limit of quantification (10Sd/*K*) was also evaluated by the above assumptions. The limit of detection (LOD) and limit of quantification (LOQ) is 0.31 and 1.05 ng mL^–1^. The precision is stated as the relative standard deviation (RSD) for two concentrations of cimetidine (7 and 60 ng mL^–1^). Their RSD were, respectively, obtained 4.1 and 3.2% for 10 replicate measurements.

The figures of merits were compared with some previously reported methodology applied for cimetidine determination (Table S1). As can be seen from the table, the proposed method using the **Zn-MOF** as a fluorescence probe displays comparable or better LOD and linear range.

### 3.7 Selectivity of the **Zn-MOF** Sensor in the Presence of Interference Species

**Figure 7:**
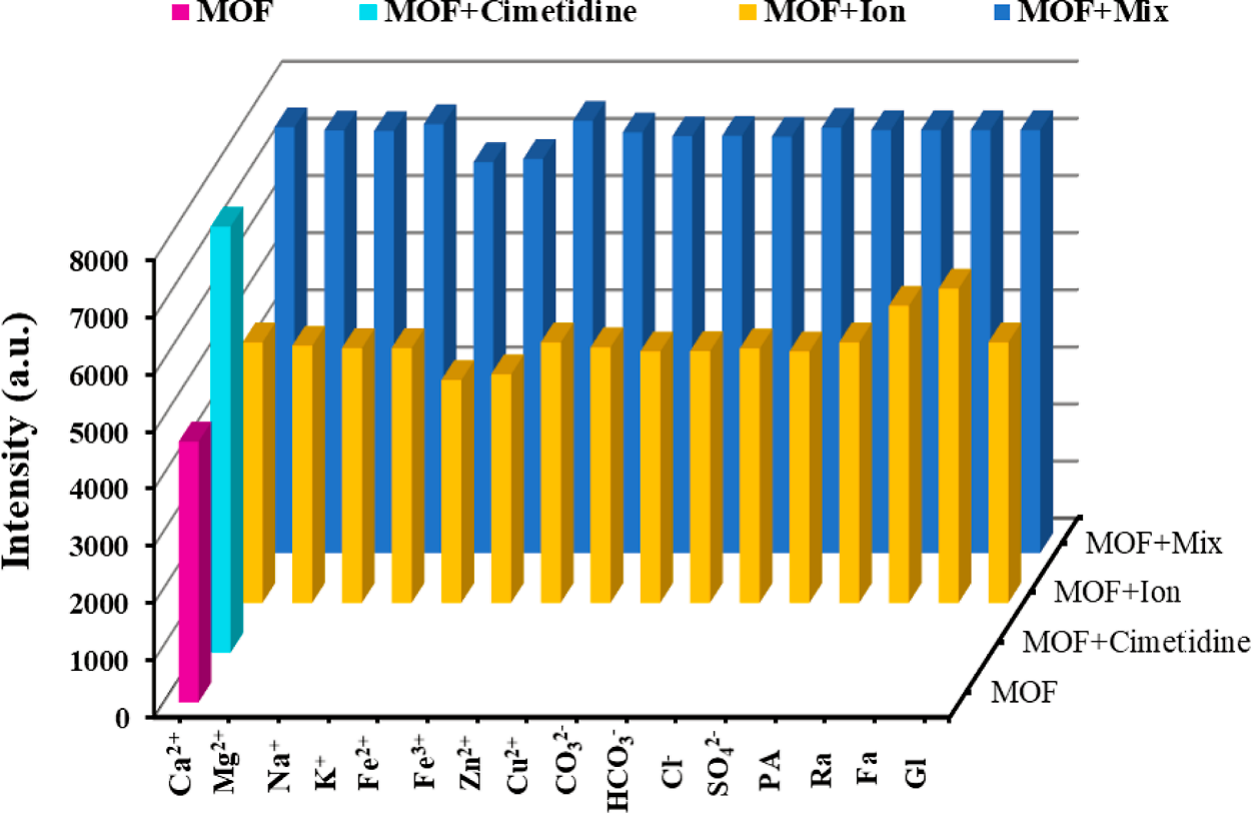
Interference investigation of coexisting species [Ca^2+^, Mg^2+^, Na^+^, K^+^, Fe^2+^, Fe^3+^, Zn^2+^, Cu^2+^, CO_3_^2–^, HCO_3_^–^, Cl^–^, SO_4_^2–^, glucose (Gl)] and drugs such as polypropanalamine (PA), ranitidine (Ra), and famotidine (Fa) on sensor response with/without cimetidine.

To check the selective sensing manner of **Zn-MOF** toward cimetidine, the experimental protocol was performed in the presence of various ions, a concomitant prescription drug (i.e., polypropanalamine), and similar structures of cimetidine (i.e., ranitidine and famotidine) alone. All these species had no impressive effect on the enhancement of fluorescence under similar experimental conditions, which propounds the selective sensing manner of **Zn-MOF** toward cimetidine. The selectivity was also examined in the presence of a mixture of drugs to investigate some competition behaviors (Figure 7). When various species were added to the **Zn-MOF** suspension, the emission intensities were not affected much even at high concentrations (200 times of 80 ng mL^–1^) but an efficacious enhancement was observed after adding cimetidine. This finding confirms the considerable selectivity.

### 3.8 Real-Sample Assay

For inspection of the reliability of the proposed sensor, the fresh plasma, urine, and commercial tablet, and ampoule samples were tested by the instruction of the proposed method. Various concentrations of cimetidine were spiked into the stated samples. The recovery was calculated for each sample after spiking the standard amounts of cimetidine under optimal conditions. The outcome data are listed in Table 3. Satisfactory recoveries in the range of 93.3–104.0% indicate that the ingredients of real samples have not interfered with cimetidine measurements at all.

**Table 3:** Analytical Results for Cimetidine Determination (*n* = 5) in Various Real Samples Using the Proposed Method at Optimal Conditions

real samples	added (ng mL^–1^)	found^a^ (ng mL^–1^)	recovery (%)
human plasma	0.0	60 ± 0.5	
	10	69 ± 0.3	98.6
	20	78 ± 0.1	97.5
urine	0.0	ND^b^	
	40	38 ± 0.5	95.0
	60	62 ± 0.6	103.3
tablet	0	50 ± 0.5	
	10	56 ± 0.8	93.3
	20	67 ± 0.7	95.7
ampoule	0	55 ± 0.1	
	10	66 ± 0.2	101.5
	20	78 ± 0.3	104.0

a *x* = *ts*/√*n* at 95% confidence (*n* = 3).

b ND: Not Detected.

To establish the accuracy evaluation, the content of cimetidine in the tablet and ampoule samples was measured through the proposed procedure. Terminal data of their analysis were estimated and compared with a labeled value of the pharmaceutical manufactory. The findings are illustrated in Table 4. As can be seen, there are no significant differences between the recommended analytical method and reported values. Hence, the proposed **Zn-MOF** sensor could be effectively applied for cimetidine assaying in biological fluids and pharmaceutical factories.

**Table 4:** Comparison of the Recommended Method for Two Real Samples Containing Cimetidine with Reported Labels of the Manufacturer^a^

real samples	reported value	found by the developed method	relative error (%)
ampule	200 mg/2 mL	205.4 ± 0.9	–2.7
tablet	200 mg/tablet	190.8 ± 0.6	–4.6

a *x* = *ts*/√*n* at 95% confidence (*n* = 3).

## 4 Conclusions

In summary, a new zinc-tetracarboxylate MOF was synthesized via in situ ligand formation by the solvothermal reaction. The synthesized MOF reveals a three-dimensional structure with open rhomboidal channels. The channels were occupied by the guest solvent molecules. The compound acts as an effective room-temperature fluorescent sensor for selective and sensitive detection of cimetidine. The fluorescence response of the sensor was enhanced in the presence of various concentrations of cimetidine (on-mode). The phenomenon was investigated by FE-SEM, DLS, and EDX mapping analyses. Based on these findings, this luminescence turn-on sensor illustrates good sensitivity and selectivity over two linear ranges of 1.0–10.0 and 20.0–80.0 ng mL^–1^, respectively. The LOD and LOQ are calculated to be 0.31 and 1.05 ng mL^–1^, respectively. Further, the proposed sensor has been successfully conducted for cimetidine detection in the human plasma, urine, commercial ampule, and tablet. The applicability of the sensor was established using the obtained good recovery percentages. Considering the simplicity of the operation methodology, the current method is recommended to be applied for typical routine analysis. This work suggests a new strategy that may open a new level of interest in MOF-based sensors for on-site and real-time analytical detection of drugs in pharmaceutical factories and medical laboratories.

## Supporting Information Available

Coordination modes of the btca^*n*–^ ligand; IR spectrum, PXRD patterns, TGA curve, and UV–vis spectra of **Zn-MOF** in different solvents; graphs of pH and reaction time effect on the intensity of **Zn-MOF** sensor; and comparison of cimetidine determination by various methods (PDF)
Details of crystallographic information (CIF)

## Supplementary Material

ao2c00874_si_001.pdf

ao2c00874_si_003.cif

## Accession Codes

CCDC 2131644 contains the supplementary crystallographic data for this paper. These data can be obtained freely via http://www.ccdc.cam.ac.uk/data_request/cif, by e-mailing data_request@ccdc.cam.ac.uk or by contacting directly the Cambridge Crystallographic Data Centre (12 Union Road, Cambridge CB2 1EZ, UK. Fax: +44 1223 336033).
