# Supplementary material for: Design of a Sensitive Fluorescent Zn-Based Metal–Organic Framework Sensor for Cimetidine Monitoring in Biological and Pharmaceutical Samples
Source: ACS Omega. 2022 Jun 21;7(26):22221–31. doi: 10.1021/acsomega.2c00874 (PMC9260946; doi:10.1021/acsomega.2c00874)
Supplement: Supplementary file 1 — ao2c00874_si_001.pdf [file ao2c00874_si_001.pdf]

Supporting Information

Design of a Sensitive Fluorescent Zn-based Metal-Organic Framework (Zn-MOF) Sensor for Cimetidine Monitoring in Biological and Pharmaceutical Samples

Zahra Afravi [a], Valiollah Nobakht [a,*], Nahid Pourreza [a], Matineh Ghomi [a], Damian Trzybiński [b], and Krzysztof Woźniak [b,*]

[a]*Department of Chemistry, Faculty of Science, Shahid Chamran University of Ahvaz, Ahvaz, Iran*

[b]*Biological and Chemical Research Centre, Department of Chemistry, University of Warsaw, Żwirki i Wigury 101, 02-089, Warszawa, Poland*

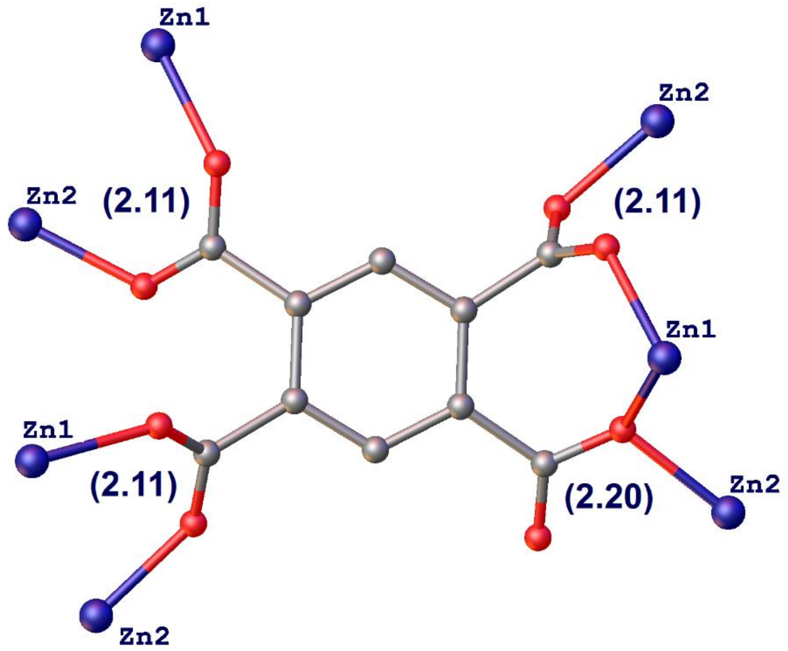

Figure S1. Three different coordination modes of $btca^{4-}$ linker in the structure of **Zn-MOF** with Harris notations.

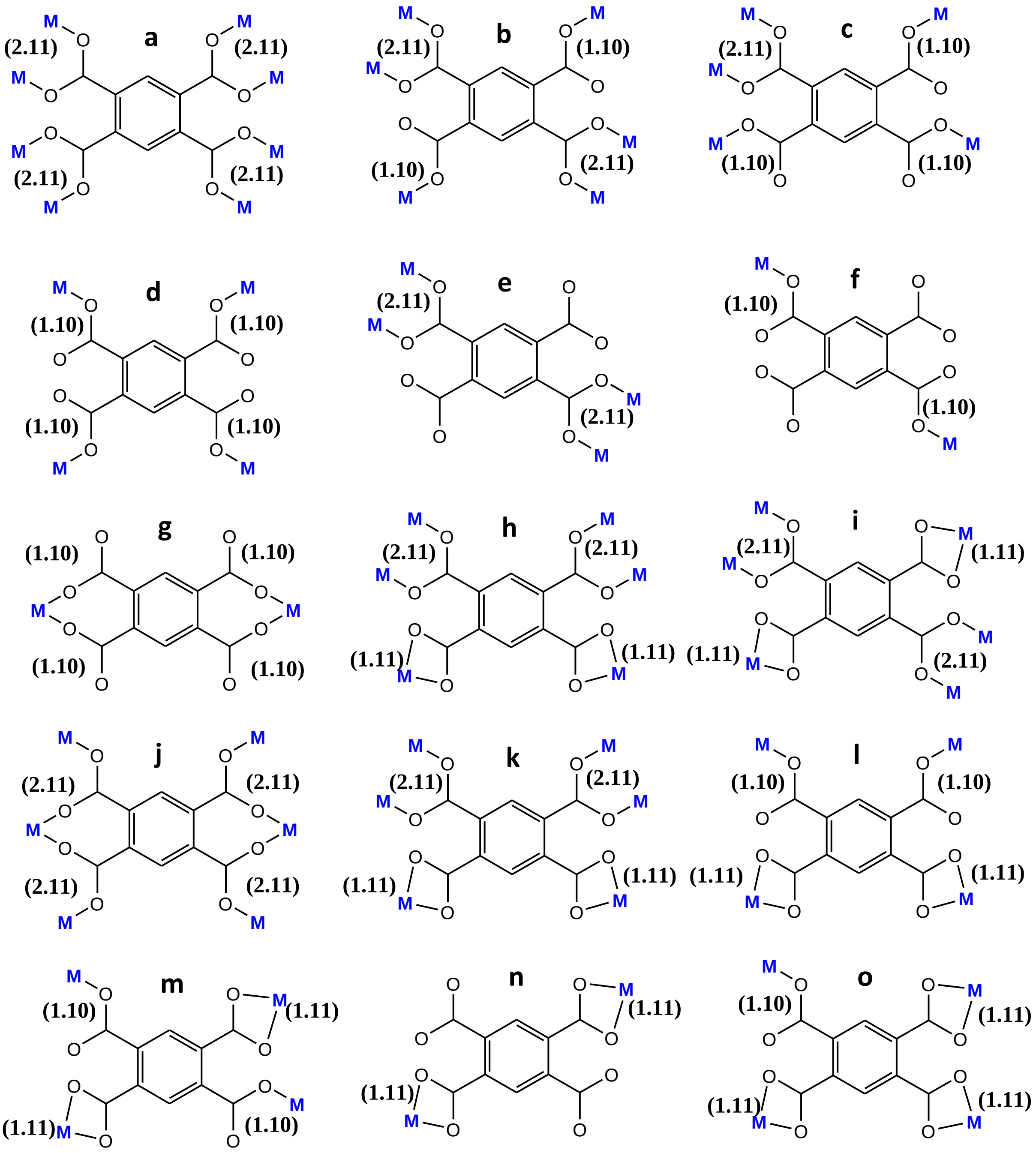
a
(2.11)
(2.11)
(2.11)
(2.11)
b
(2.11)
(1.10)
(1.10)
(2.11)
c
(2.11)
(1.10)
(1.10)
(1.10)
d
(1.10)
(1.10)
(1.10)
(1.10)
e
(2.11)
(2.11)
f
(1.10)
(1.10)
g
(1.10)
(1.10)
(1.10)
(1.10)
h
(2.11)
(2.11)
(1.11)
(1.11)
i
(2.11)
(1.11)
(1.11)
(2.11)
j
(2.11)
(2.11)
(2.11)
(2.11)
k
(2.11)
(2.11)
(1.11)
(1.11)
l
(1.10)
(1.10)
(1.11)
(1.11)
m
(1.10)
(1.11)
(1.11)
(1.10)
n
(1.11)
(1.11)
o
(1.10)
(1.11)
(1.11)
(1.11)
M
O

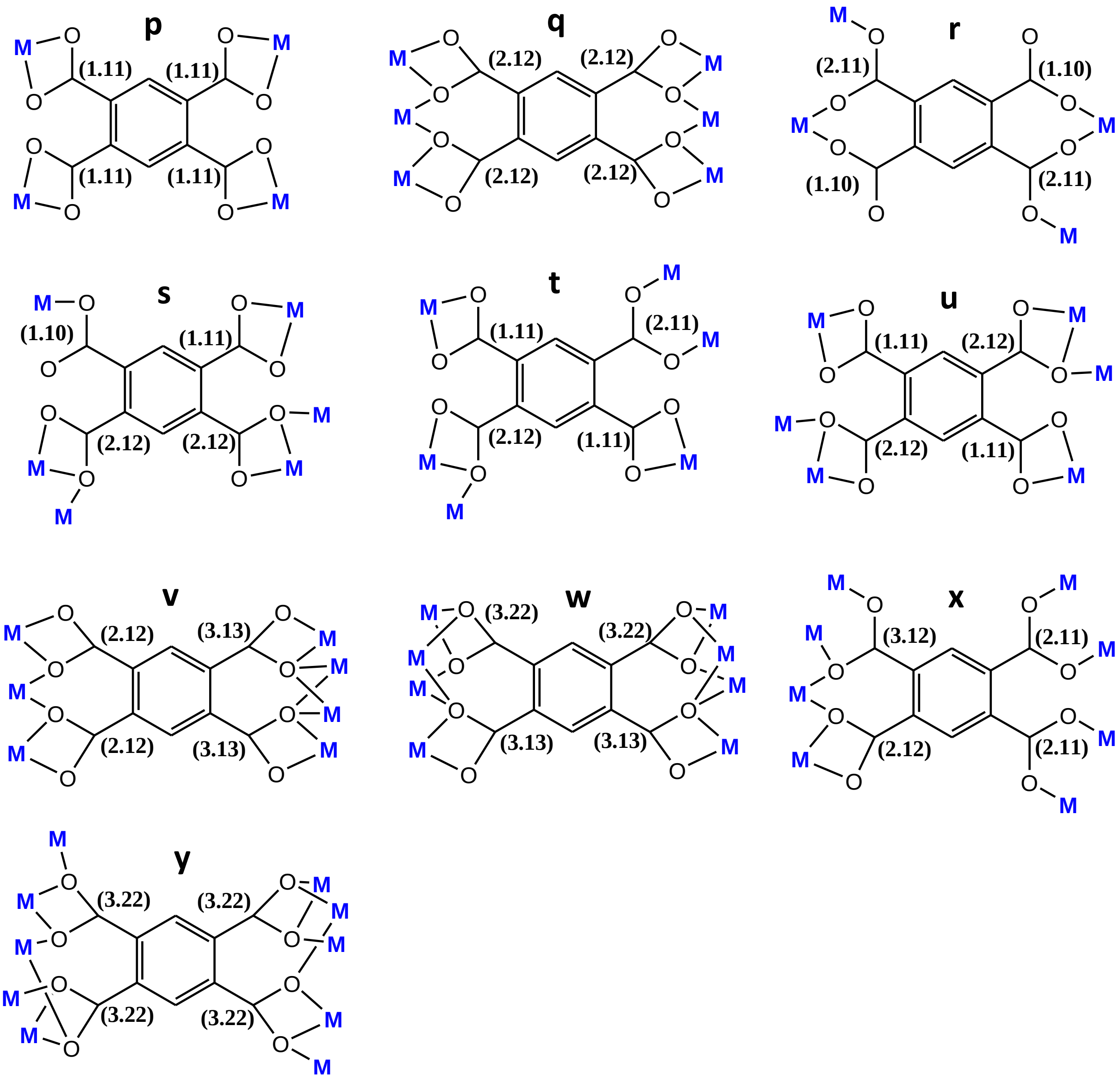

Figure S2. Some of the common reported coordination modes of btca^{n-} with Harris notations.

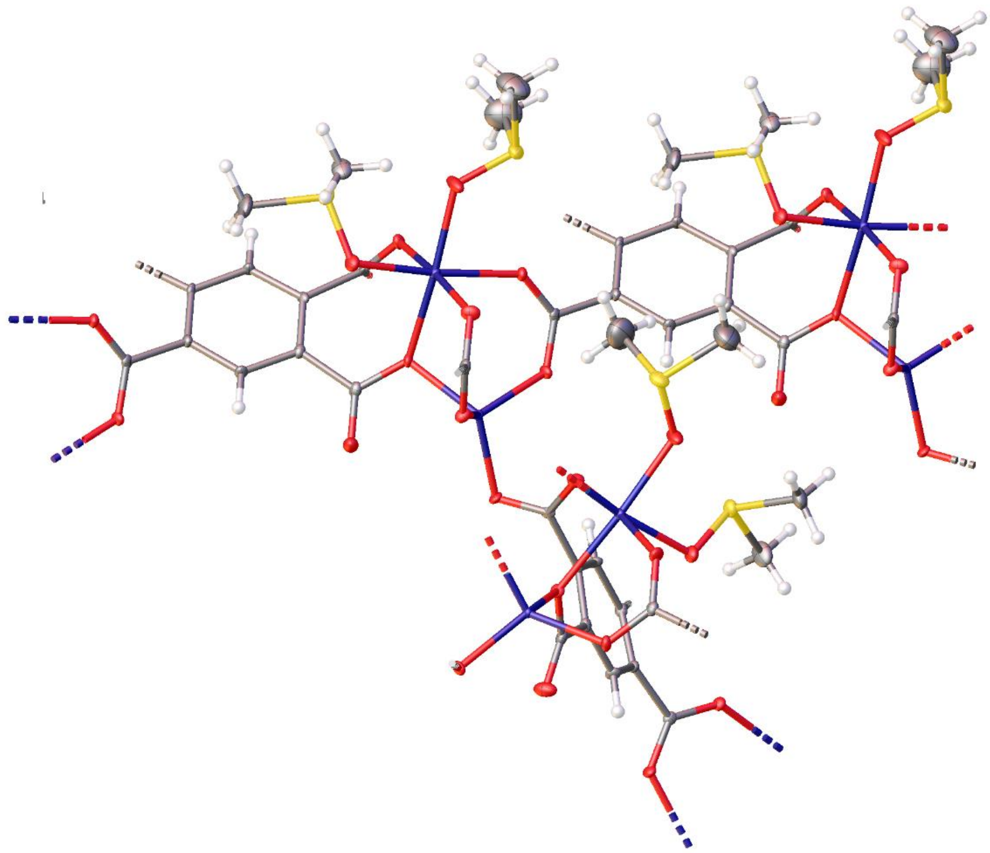

Figure S3. Connection of three repeat units in the 3D crystal structure.

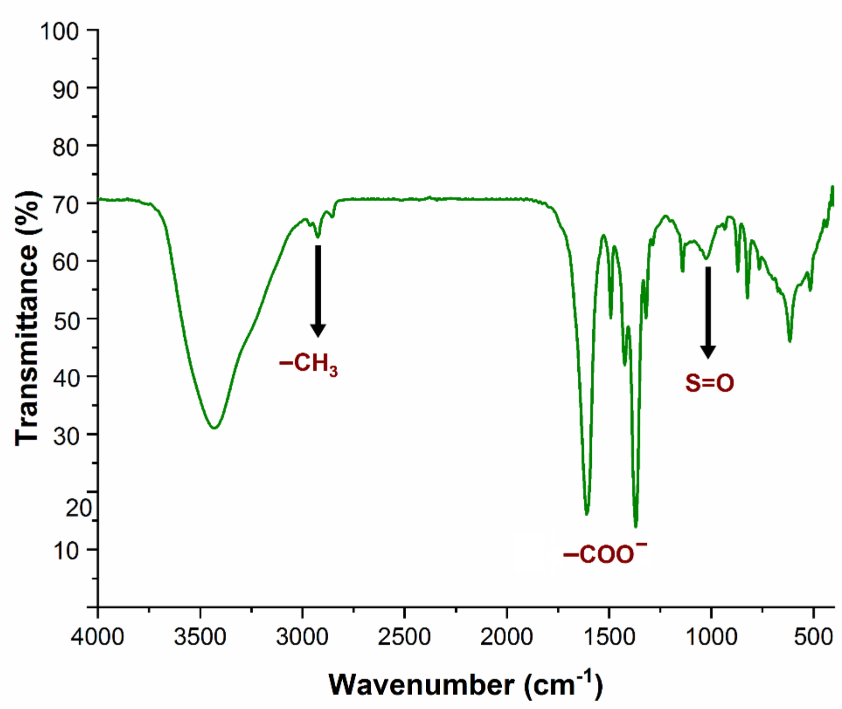

Figure S4. FT-IR spectrum of **Zn-MOF**.

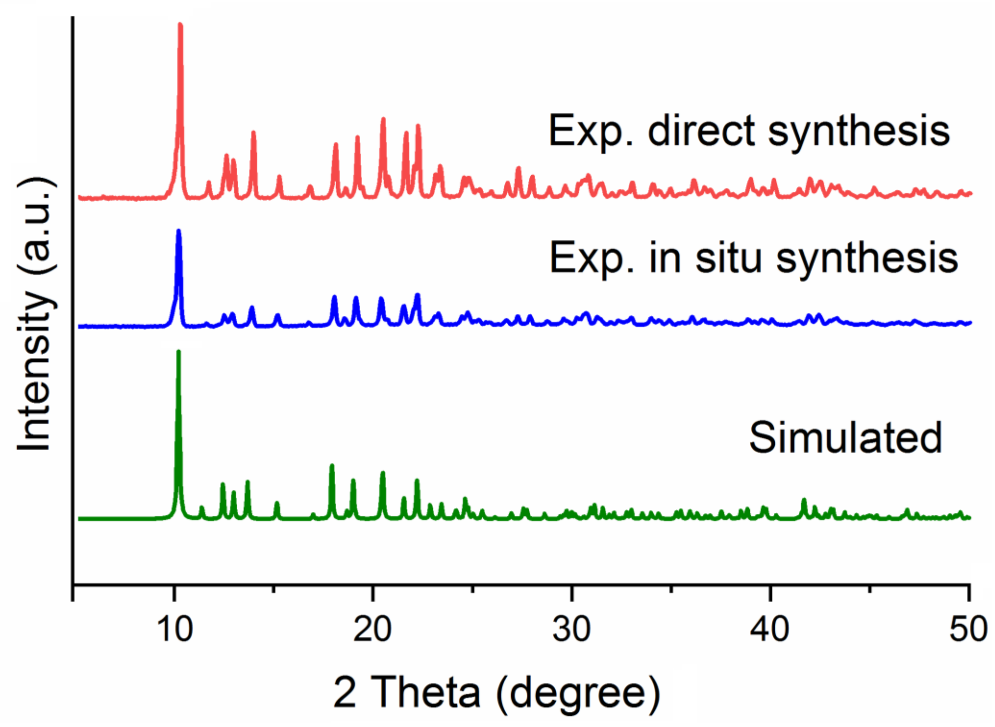

Figure S5. Comparison of the experimental PXRD pattern for **Zn-MOF** in direct (red) and in-situ (blue) synthesis with the pattern simulated from the single crystal data (green).

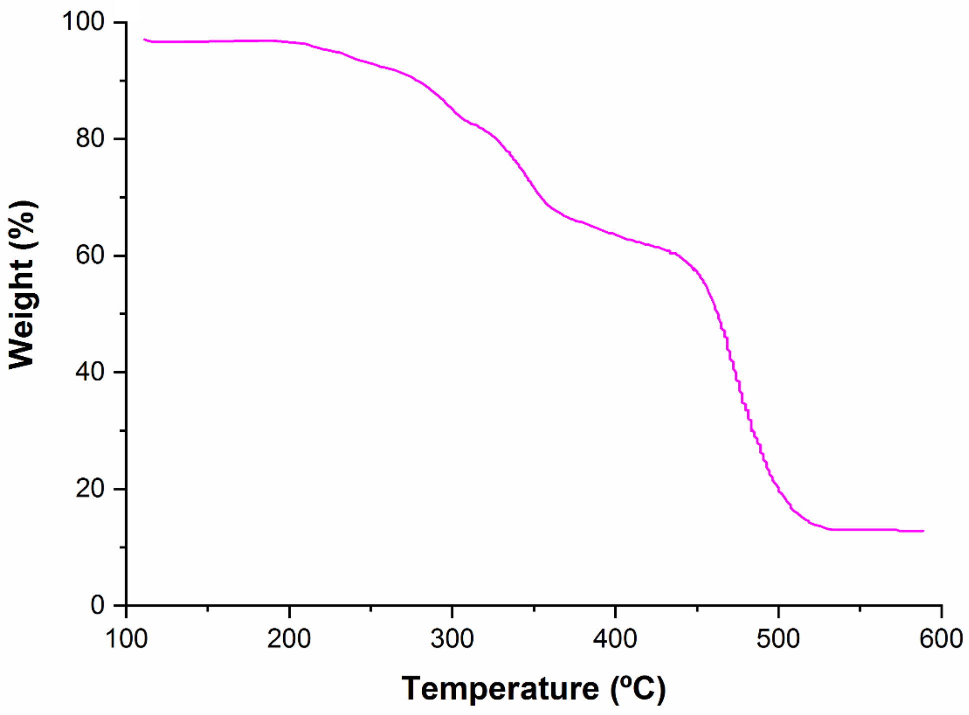

Figure S6. Thermogravimetric (TG) curve recorded for **Zn-MOF**.

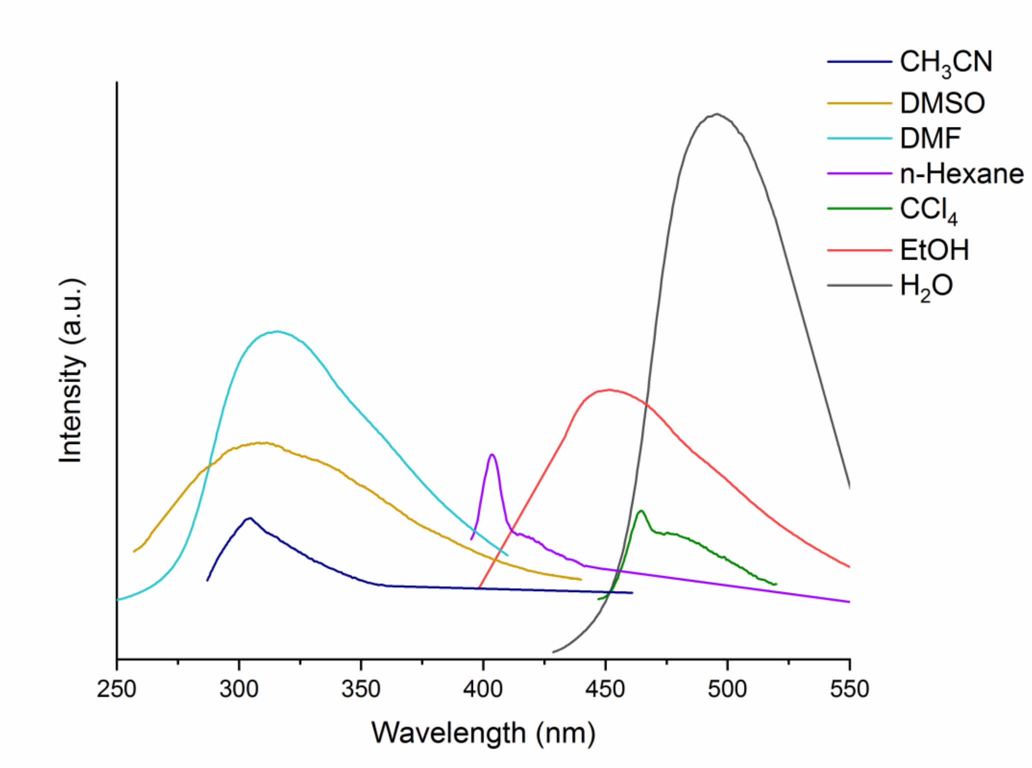

Figure S7. UV-Vis spectra of dispersed **Zn-MOF** in different solvents with various polarities.

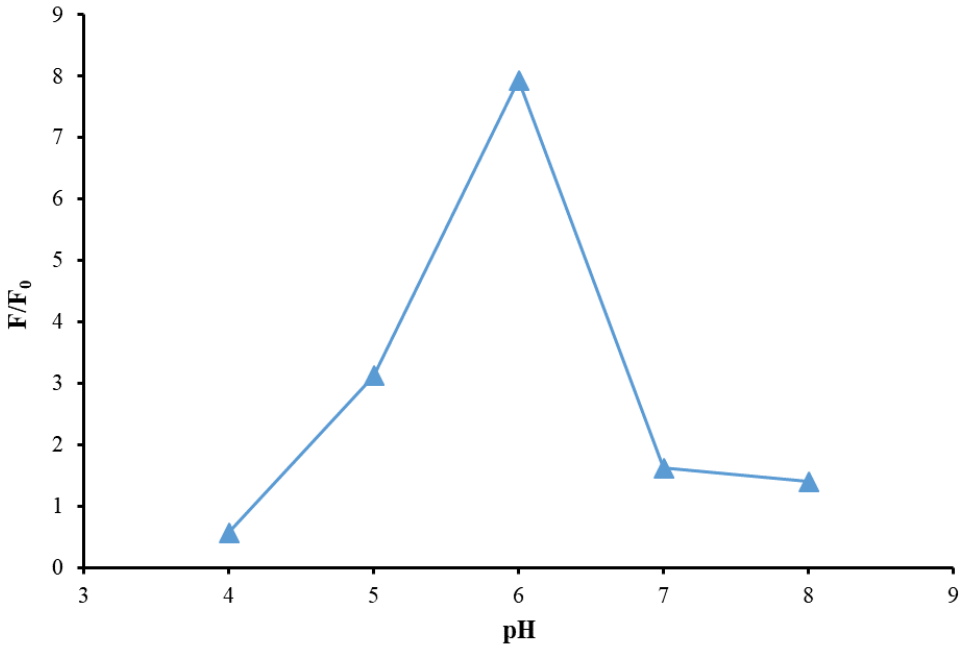

Figure S8. The effect of various pH on the output signal of **Zn-MOF** sensor.

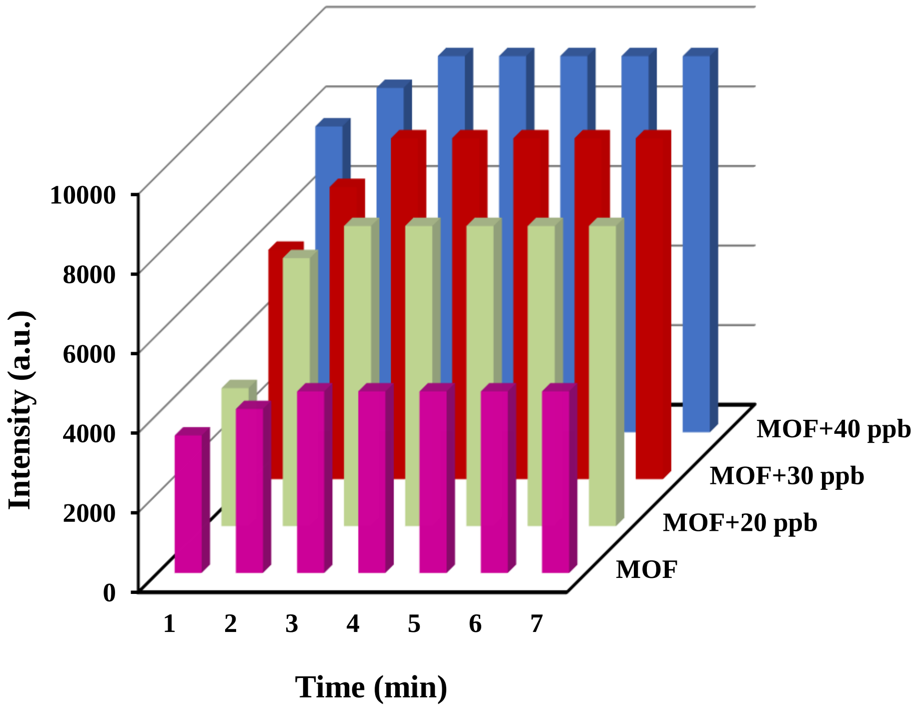

Figure S9. The effect of reaction time on the signal of **Zn-MOF** in the presence of cimetidine.

Table S1. Comparison of cimetidine determination *via* the proposed method and other methodologies.

Method	Linear range ($\mu g\ mL^{-1}$)	LOD ($\mu g\ mL^{-1}$)	RSD (%)	Ref
Spectrophotometry	0.01–0.5	0.006	N.R.[a]	1
Spectrophotometry	8–30	1.220	0.8	2
Spectrophotometry	0.5–15	0.222	1.158–2.003	3
HPLC-UV	3–500	0.5	0.2–13.6	4
HPLC-MS/MS	0.025–6	N.R.	0.59–6.85	5
Fluorimetry	0.04–1.9	0.013–0.030	N.R.	6
Electrochemistry	$2.52–2.52 \times 10^3$	1.261	N.R.	7
Fluorimetry	0.001–0.08		3.2–4.1	Current work

[a] **Not Reported**

References

(1) Wei, X.; Du, L.; Wang, L.; Wei, C.; Jiang, Z., Spectrophotometric Determination of Cimetidine through Charge-transfer Reaction with 1, 5-Dichloroanthraquinone as a π-Electron Acceptor, *Chin. J. Chem.*, 27 (**2009**) 1624-1628.

(2) Darwish, I.; Hussein, S.; Mahmoud, A.; Hassan, A., A sensitive Spectrophotometric method for the determination of H2-receptor antagonists by means of N-bromosuccinimide and *p*-aminophenol, *Acta Pharm.*, 58 (**2008**) 87.

(3) Dikran, S.; Mohammed, A.; Al-Jumaily, A., Univariate and simplex optimization for the spectrophotometric determination of cimetidine and erythromycin ethylsuccinate drugs using bromothymol blue via ion-pair formation, *Ibn AL-Haitham Journal For Pure and Applied Science*, 23 (**2017**) 170-189.

(4) Ashiru, D.A.; Patel, R.; Basit, A.W., Simple and universal HPLC-UV method to determine cimetidine, ranitidine, famotidine and nizatidine in urine: Application to the analysis of ranitidine and its metabolites in human volunteers, *J. Chromatogr. B: Biomed. Sci. Appl.*, 860 (**2007**) 235-240.

(5) de Sousa, C.E.M.; Tabosa, M.A.M.; de Lima, E.N.; de Souza Filho, J.; Homero, e.; Bedor, D.C.; Galindo, e.; Leal, L.B.; de Santana, D.P., Development of a simple and rapid method for the determination of cimetidine in human plasma by high performance liquid chromatography-mass spectrometry (HPLC-MS/MS): Application to a bioequivalence study, *Afr. J. Pharm. Pharmacol.*, 8 (**2014**) 1156-1163.

(6) Chang, Y.-X.; Qiu, Y.-Q.; Du, L.-M.; Li, C.-F.; Guo, M., Determination of ranitidine, nizatidine, and cimetidine by a sensitive fluorescent probe, *Analyst*, 136 (**2011**) 4168-4173.

(7) Shamsipur, M.; Jalali, F.; Haghgoo, S., Preparation of a cimetidine ion-selective electrode and its application to pharmaceutical analysis, *J. Pharm. Biomed. Anal.*, 27 (**2002**) 867-872.
